# Abstract of Proceedings of the Buffalo Medical Association

**Published:** 1866-06

**Authors:** T. M. Johnson

**Affiliations:** Sec’y.


ART. II.—*Abstract of Proceedings of the Buffalo Medical Association.*

TUESDAY EVENING, April 3d, 1866.

The Association was called to order at the usual hour by the retiring President, Dr. RING. Present—Drs. Ring, Rochester, Gay, Gould, Cronyn, Congar, Tafft and Johnson.

The report of the proceedings of the last meeting was read by the Secretary, and adopted.

Dr. Gay moved that a committee of two be appointed to conduct the President elect to the chair. Carried.

The President appointed Drs. Gay and Congar such committee, by whom the President, Dr. William Gould, was conducted to the chair, and delivered the following

### INAUGURAL ADDRESS.

Gentlemen of the Association:

Before entering upon the duties devolving upon me as your presiding officer, I propose to make a few remarks, which, I trust, will not be considered out of place on this occasion. But first permit me to return you my most sincere thanks for the honor conferred upon me. I deem it no empty honor to be elected to preside over this Association. It is all the more gratifying to my feelings because entirely unsought for. An abler and more experienced member might with great propriety have been selected to preside over your deliberations. As I shall occupy the chair, not from my own volition, but from your choice, I shall endeavor, to the best of my ability, to discharge its duties.

Of the past it is unnecessary to speak, more than to notice the gratifying circumstance, that in a financial view, the Association is in a sound and healthy condition. For this agreeable state of things much credit is due our efficient financial officer, Dr. Lockwood.

The non-attendance of members during the year just closed is to be regretted. Individual absence no doubt is often unavoidable from the nature of our occupation, and very often indeed from circumstances over which we have no control. But I fear we have not always this very proper and valid excuse. Our Society numbers about forty members, and we often have a bare quorum at our regular monthly meetings. I have thought that societies were much more prosperous, and better attended, when they meet weekly instead of monthly. It may be said that if our meetings are not well attended monthly, it would be worse if we met every week.

My own opinion is that the oftener we get together the more interest would be manifested in our deliberations. Then, again, by frequent meeting and contact, the younger and more diffident members would be encouraged to take part in the report and discussion of the various subjects brought before the Association. However I merely advance the suggestion that we meet once a week during the summer months. I would also suggest the propriety, if we have the necessary funds in the treasury, of having the Constitution and By-Laws of the Association printed in convenient form for distribution.

If we except variolæ, so prevalent the past fall and winter, our city for years has been free from epidemic disease, at least to any extent. Consequently our professional labors have been comparatively light and free from danger. But we are forced to look forward to the coming season with anxious solicitude. Asiatic cholera has again taken up its westward march, and has already reached our shores. We can hardly expect to escape its devastations should it once leave the metropolis and follow, as it undoubtedly would, the great lines of communication westward which traverse our State. Should it come, it devolves upon the medical fraternity to present an unbroken front, and do battle with the fell destroyer. ' A season of ordinary health," it is said, "is to the physician like a time of peace to the soldier; but the visitation of a fearful epidemic is the war in which he goes forth to battle, and to the struggle with death, that he may save others, and perhaps perish himself in saving them."

MEASURES FOR PUBLIC HEALTH.

Already measures have been inaugurated to unite our efforts with that of the Board of Health, and propositions made to divide the city into districts, and to provide the poor, and all others who by force of circumstances may require it, prompt medical attendance. This is all very well, so far as it goes, but it devolves upon us to see that this and all other hygienic measures proposed should be carried into immediate effect. If we can have thorough organization and prompt, efficient action on the part of the Board, the battle will be half won, and the usual panic on such alarming occasions as the visit of epidemic cholera may be avoided, and our citizens, by enjoying the fullest confidence, will be much less liable to take the disease.

OUR BOARD OF HEALTH.

It may be considered as trenching upon forbidden ground, to speak of the organization of the Board of Health as it now exists.

My views are that the commissioners should at all times be residents of the city proper, and the office entirely separated from political influence. The present presiding officer of the Board is very appropriately a physician, but could he be expected to leave home, his family, and his patients, to spend his time, during the summer especially, in the city, exerting his energies, perhaps sacrificing his life, as others have done heretofore, for the paltry consideration of one hundred and fifty dollars per year? We know that during previous cholera seasons the duties of the Board of Health have been both laborious and dangerous. In 1849, the President of the Board fell a victim, while with a zeal worthy a better fate, he worked night and day in behalf of suffering humanity. Many of you, as well as myself, also remember the labors of McArthur, and our lamented friend and brother, Dr. Newman, during the epidemic of 1852–4.

A BOARD BORN TO BLUSH UNSEEN.

With the present Board as men I have no objection; it is with regard to their location that I take exception. For instanee, one lives at Black Rock dam, one out in the Thirteenth Ward, and the third Heaven knows where, for his name and residence is not to be found in the directory of this or former years, which fact may be taken as an indication that he may or may not live anywhere in the city limits. I do not object to the politicians in the outside territory claiming and obtaining a full share of all political offices, except that of the Health Department. That, I claim, should be within the old city limits, and as centrally located as possible; and I also believe it would be most judicious if medical men were to be selected as commissioners. If united action could be inaugurated among ourselves, and the subject properly presented to the public, I have no doubt all that is claimed for the profession would be generously conceded.

SOURCES OF DISEASE.

In all large cities there are certain sources which generate disease and feed epidemics, requiring the most assiduous care, not only by the Board of Health, but of physicians. The most prominent may be classified or designated under two heads: that of food for the proper nourishment of the body, and shelter to protect from the inclemencies of the weather; or, to be more explicit, I

may say markets and tenement houses. In the markets unhealthy meats and stale vegetables are dealt out to unsuspecting customers by unprincipled dealers, the consumption of which are often productive of disease and death. But a few days since a case came under my observation, where a whole family were made sick from eating veal purchased at one of our markets. It was described as fair to the eye, but proved too much for the digestive organs of the partakers. Severe cholera morbus and diarrhœa followed its use. Very similar results followed the eating of pork purchased at the same market by another family. As conservators of the public health, we should on every possible occasion warn the people to be careful and judicious in selecting their meat and vegetables, as thereby much sickness and suffering, and even death, may be avoided.

### TENEMENT HOUSES.

In regard to the inmates of tenement houses, how true the saying, "that one-half the world know not how the other half live." To this rule we may justly claim exception. We do have occasion to see and know more of these dark abodes of misery than any other class of community. What sad spectacles are often presented to our view in those filthy abodes of vice and poverty! My individual experience would fill a volume if rehearsed, and yet many of my professional brethren could, no doubt, go far beyond me in such revelations.

### AN OFFICIAL VISIT.

I once invited a gentlemen who had a voice in auditing my accounts for attending the poor, and was an incessant grumbler at my charges, to go with me in my rounds. Of course I took good care that he should see the dark side of the picture, and took him to the sickest patients and into the most filthy apartments, the foul odors of which outdid Shakspeare's "compound of most villainous smells." They were too much for his delicate olfactories. The next auditing day proved it to be the most profitable day's work I had done during the year, for I got a more liberal allowance and with less grumbling. Those of our citizens comfortably housed, and whose families are bountifully fed, realize but little of what transpires in the dark, damp abodes of the poor, where the bright sunlight never penetrates, and where a breath of pure air is never enjoyed. Filth and foul odors are not all that meet us there, nor do we always escape with what is only offensive to sight and smell. Such abodes furnish abundant food for cholera. It is there pestilence fattens, and from there gathers force to make its forays upon the better class of our citizens.

### WORK FOR THE BOARD.

Here, then, is a large field for the active efforts of the Board of Health, and for us. In what way these nuisances can be best abated when an epidemic is impending, is a difficult problem to solve. If they cannot be annihilated, they should be certainly regulated as far as possible. In what particular way the inhabitants of these dens can be disposed of, in case we have a visitation of cholera, is another important question. If driven from one quarter by the Board of Health, it is only to take shelter in another. They cannot live in the streets, nor have our asylums sufficient capacity for their accommodation. Let us ponder well upon this subject, and, I trust, that before danger is fully upon us, some philanthropist will inaugurate measures which will free our beautiful city from its plague spots. Let us remember that thought begets action, and that but for the fertile mind and indomitable perseverance of a Wilcox, our city would not to-day have a General Hospital in successful operation.

### A WORD IN SEASON.

In the mean time let us, as physicians, look well to those under our charge; and in view of the approaching danger, warn them against all exciting and predisposing causes of cholera, for no doubt in a great measure it is a preventable disease; and even in its earliest stage can be easily arrested. Let them avoid sudden exposures of temperature, severe laborious exercise, all excesses in eating and drinking, damp and unhealthy dwellings, mental anxiety and fear, for of such comes death when cholera prevails. In order also to guard our citizens from the imposition of quack nostrums always in the market and so extensively advertised in the papers, let us provide every family under our care with a proper remedy, with directions for instant use in case of an attack, for a physician cannot always be found at the moment he is wanted,

An individual or a family thus protected and provided for feels the same courage and confidence in regard to cholera, that a man feels with a loaded revolver in his hand when he meets a highwayman in the street, or finds a burglar in his house.

### DATA NEEDED.

It is to be regretted that for several years we have had no published annual report of the Health Physician, if I do not mistake since that made by Dr. Strong in 1859. What reason the Board of Health may have for suppressing or neglecting to publish this document, has not to my knowledge transpired. Whether it is from inefficiency or a desire to save the expense of printing on their part, is left to conjecture. Why the department should resolve itself into a close corporation and keep the reports from the public eye, needs, and we have a right to demand, an explanation. Whatever the reason may be, it is wrong, and the Health Physician in justice to himself should insist upon its publication. Aside from the general reader, mortuary reports contain matters of special interest to the profession.

### AN ABUSE TO BE CORRECTED.

In this connection permit me to refer to the matter of certificates of cause of death. Many of us no doubt are careless and dilatory in writing them out. This should be promptly corrected by those of us heretofore remiss. They should always be made with care, and left at the house or at the physician's office, so that they can be had when called for. When this is done our duty in the case is fully performed. The city ordinances require us to furnish the certificate forthwith, under a penalty of $25 for neglect. They also forbid the burial, or removal, of any person dying in the city without the sexton first obtaing a permit from the City Clerk, under the same penalty. I presume it is a well known fact to every one present, that the sextons pay little regard to this requirement; and with this laxity on the part of those entrusted with the enforcement of the laws, what reliability can be placed upon the record of mortality? When a sexton takes a body to the cemetery for burial, without a permit, he should be refused admission. Administer a lesson of this nature, and he would not be likely to repeat the experiment. The keepers of all the cemeteries should be held strictly accountable as well as the sextons and the evil would be cured.

Gentlemen:—I am aware that this is a Medical Association and not a Board of Health. The subject, I know, properly belongs to the latter, yet I have taken the liberty of introducing it here because it is what we are all interested in, and if it be the means of reformation of the faults referred to, the time will not be spent in vain, and I trust in a city like ours more regard will in future be paid to these important matters. Whatever the faults of others may be, let us enjoin it upon ourselves to do our whole duty, not only in the matters referred to, but in everything pertaining to our noble and self-sacrificing profession, so that should we fall by the way, and before our allotted time, we may enjoy the consoling reflection that we have faithfully discharged our obligations, not only to God "in whom we live and have our being," but to our country, society, and ourselves.

Dr. Gay moved that the thanks of the Association be tendered to Dr. Gould for his address, and that a copy be requested for publication in the *Buffalo Medical Journal* and in the daily papers. Carried unanimously.

Dr. Rochester stated that as Chairman of the Sanitary Committee, he had become acquainted with the manner in which the duties of the Board of Health were performed, and had become disgusted. He could get them to do nothing and had become entirely discouraged, and was glad that the President had spoken so plainly and pointedly upon the subject.

Miscellaneous business being in order, Dr. Congar moved that Dr. Andrew Camerling be invited to participate in the exercises of the Association until he becomes eligible to membership. Carried.

The prevailing diseases were reported to be pneumonia, diarrhœa and typhoid fever.

Dr. Rochester reported the following case:—A man about twenty-five years old who had usually enjoyed good health, was attacked with severe pain in the ear. Soon after the attack he left the house and wandered about the streets, and showed by his actions and conversation that he was insane. When the doctor first saw him he found purulent discharge from the left ear, violent pain, sometimes saw double, the eye large and bulging, and considerable delirium, but no paralysis. He had passed two or three sleepless nights and days. Injected the fourth of a grain of sulphate of morphia, after which the patient slept ten hours and then felt better. Twenty-four hours after was not so well, the pain having increased. Injected a fourth of a grain of sulphate of morphia. About two hours after this he went to sleep and soon became comatose, and so remained until he died. First supposed he had nothing but otitis. Post mortem examination showed no disease of the petrous portion of the temporal bone. There was diffusion of four or five ounces of pus about the medulla oblongata. Considerable pus was also found covering most of the surface of the base of the brain and infused into the arachnoid membrane. It was undoubtedly disease of the base of the medulla spinalis. Never have met with inflammation of the brain with otitis.

Adjourned. T. M. Johnson, Sec'y.

